# Identification and evaluation of network modules for the prognosis of basal-like breast cancer

**DOI:** 10.18632/oncotarget.4034

**Published:** 2015-05-08

**Authors:** Robin M. Hallett, Jessica G. Cockburn, Brian Li, Anna Dvorkin-Gheva, John A. Hassell, Anita Bane

**Affiliations:** ^1^ Department of Biochemistry and Biomedical Sciences, Centre for Functional Genomics, McMaster University, Hamilton, Ontario, Canada; ^2^ Department of Oncology, McMaster University, Hamilton, Ontario, Canada

**Keywords:** basal-like breast cancer, gene expression, prognosis, networks

## Abstract

**Purpose:**

Basal-like breast cancer (BLBC) is a molecular subtype of breast cancer associated with poor clinical outcome, although some patients with BLBC experience long-term survival. Apart from nodal status, current clinical/histopathological variables show little capacity to identify BLBC patients at either high- or low-risk of disease recurrence. Accordingly, we sought to develop a network based genomic predictor for predicting the outcome of patients with BLBC.

**Experimental Design:**

We performed network analysis on global gene expression profiling data of BLBCs, and identified BLBC network modules associated with AP-1 transcription, G-protein coupled receptors, and T-, B-, and NK-cells that are significant predictors of BLBC patient survival.

**Results:**

In gene expression and tissue microarray (TMA) validation cohorts of 210 and 102 BLBC patients, respectively, the identified network modules were robustly associated with patient outcome. In the gene expression validation cohort, the Kaplan-Meier estimate for 10-year survival in the low-risk group was 90%, whereas in the high-risk group it was a 56%. In the TMA cohort, the Kaplan-Meier estimate for 10-year survival in the low-risk group was 98%, whereas in the high-risk group it was 71%.

**Conclusions:**

The capacity to distinguish between patients with BLBC at high- or low-risk of recurrence at the time of diagnosis could permit timely intervention with more aggressive therapeutic regimens in those patients predicted to be high-risk, and to avoid such therapy in low-risk patients.

## INTRODUCTION

Prognostic stratification of breast cancer patients is traditionally based on a variety of factors such as tumor size, grade, hormone receptor status, HER2 status, lympho-vascular space invasion and lymph node involvement [1, 2]. However, the recent development of various whole genome analysis technologies has provided new tools for the molecular classification of breast cancer and directly contributed to the development of several genomic based predictors including a 21-gene, 70-gene, 76-gene, 77-gene genomic grade profile, 50-gene subtype, wound response signature and a ‘stemness’ signature, among others [3-10].

Basal-like breast cancer (BLBC) was first identified as a subtype of breast cancer in 2000, based on gene expression profiling experiments conducted by Perou and colleagues [11]. Several clinical reports demonstrate that BLBCs are associated with an increased risk of developing distant metastasis, shorter survival and increased mortality [12-14]. Detailed reports on the prognosis of BLBC suggest that patients with BLBCs experience high relapse rates within the first 3-5 years following diagnosis. After this period the recurrence risk rapidly declines such that over the long term BLBC patients have outcomes similar to those of patients with luminal A disease [15-18]. Hence, these findings demonstrate that patients with BLBCs can be stratified into two clinically distinct groups; those at high-risk of early recurrence and death, and those at low-risk of such an outcome and hence likely to experience long term survival.

Whereas several genomic based predictors exist to predict breast cancer patient outcome, their prognostic value appears to be mostly derived from their capacity to measure expression of genes associated with proliferation and ER status [19, 20]. Because BLBCs represent ER negative and highly proliferative tumors, existing predictors uniformly identify such patients as being at high-risk of recurrence. To overcome these challenges, we and others have focused on identifying genomic based predictors of outcome in ER negative (ER-), triple negative (ER-, PR-, HER2-) or BLBCs specifically [20-24]. However, robust methods of distinguishing between BLBC patients likely to experience either good or poor outcome has proved particularly challenging. Here, we report the identification and validation of network modules for predicting BLBC patient outcome.

## RESULTS

### Identifying BLBC outcome-associated network modules

**Figure 1:**
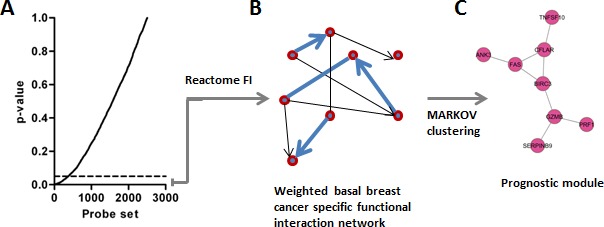
Strategy implemented to identify BLBC modules **A.** Univariate Cox-regression identifies 372 outcome-associated probe sets. **B.** Probe sets are mapped onto the Reactome network and edges are weighted based on expression correlation between nodes (genes). **C.** The weighted network is clustered and network modules are identified (*n* = 7 Pearson correlation > 0.25).

We sought to identify gene networks that might be useful to predict outcome in patients with BLBC. Briefly, we compiled gene expression profiles from 5 independent datasets, which represent non-redundant tumor samples, and for which clinical follow-up data was available (Supplementary Table 1). Together, these datasets represented 995 tumors, of which 134 were of the BLBC molecular subtype, henceforth referred to as the ‘training’ set. To identify probe sets associated with outcome, we completed univariate Cox-regression analyses for the top 2,500 most variably expressed probe sets present on the microarrays, which identified 372 probe sets significantly associated with outcome (Figure 1A, *P* < 0.05). Whenever possible, we used disease free survival (DFS) as the clinical endpoint for this analysis, although in some cases distant metastasis free survival was used. The genes represented by the 372 probe sets were then mapped as nodes onto a previously described highly reliable human functional interaction network [25]. Pearson correlation coefficients (for gene expression) were calculated for all interacting gene pairs, and assigned as ‘edges’ to this network [26] (Figure 1B). Finally, the network was clustered using MCL (Markov clustering), to identify candidate interaction modules associated with outcome (Figure 1C). Hence, each module comprises sets of genes that are topologically close in the un-weighted human functional interaction network, and also display highly co-ordinated expression in BLBC.

**Figure 2:**
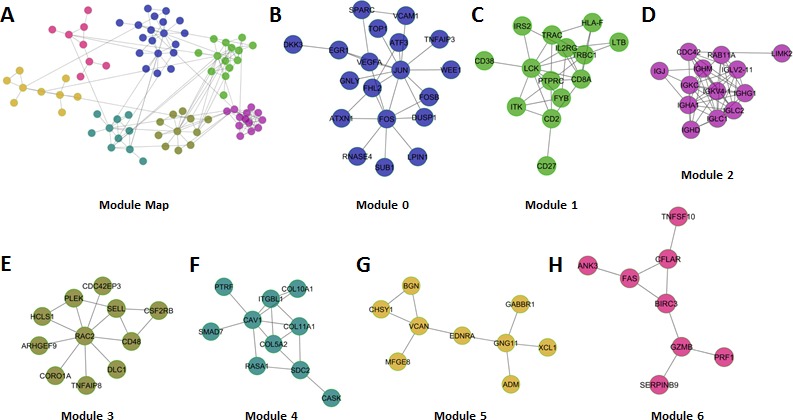
Overview of BLBC network modules **A.** Global view of the 7 network modules (circles represent nodes (Genes) and gray lines represent edges). **B-H.** Each of the individual modules is presented.

We identified 7 modules that each comprised 8 or more nodes (genes) that displayed an average Pearson correlation of at least 0.25 based on expression. Each module was numbered from 0 – 6 in decreasing module size (Figure 2A-2H). Based on the expression of the genes comprising each module, we calculated a module index that represented the difference in mean (geometric) expression between poor and good prognosis genes. Univariate Cox regression analysis of the individual module indices revealed that each module was robustly associated with patient outcome (Table 1, Hazard Ratios [HR] per unit increase in module index ranged from 1.6 - 2.3; *P-*values ranged from 0.0068 to 0.000014). A combination index (BLBC modules), representing the mean of the 7 individual module indices was a stronger predictor of patient outcome than any of the individual modules suggesting that in sum, the modules comprehensively measure the biological programs that drive patient outcome (Table 1, HR, 3.0; *P* = 0.000041). We also observed that modules generally did not comprise mixtures of good and poor prognosis genes, but rather were highly enriched for either good or poor prognosis genes. Modules 1, 2, 3 and 6 were enriched in genes whose expression was associated with good outcome, whereas modules 0, 4 and 5, were enriched in genes whose expression was associated with poor outcome.

**Table 1:** Summary of BLBC modules based survival analysis in training and validation patient cohorts

	Training		Validation	
	Hazard Ratio	*P*-value	Hazard Ratio	*P*-value
Module 0	2.3	1.40E-06	1	1.00E-04
Module 1	1.6	0.0046	2.7	2.90E-07
Module 2	1.6	0.0068	2.2	2.50E-06
Module 3	1.7	0.0016	2.5	9.00E-07
Module 4	1.7	0.0019	1.3	0.10
Module 5	2.1	1.40E-06	1.9	3.40E-05
Module 6	2.0	0.0002	2.2	8.00E-06
Combination	3.0	4.10E-06	3.1	2.10E-07

To assess the robustness of the modules, we compiled a validation cohort that comprised breast tumor sample profiles from additional independent datasets, henceforth referred to as the validation set (Supplementary Table 2). Together these datasets represent an additional 894 non-redundant tumor samples, of which 211 belonged to the BLBC molecular subtype, and for which clinical follow-up data was also available. Using univariate Cox regression to measure the relationship between the individual module indices and patient survival, we found that modules 0, 1, 2, 3, 5 and 6 were all significant predictors of patient outcome (Table 1; HR 1.9-2.7; *P-*values ranged from 0.0001 to 0.000009). The module 4 index trended as a predictor of patient outcome, but did not reach statistical significance (Table 1; HR 1.3; *P* = 0.10). As we observed with the training data, the BLBC modules score representing the mean of the 7 individual module indices was a superior predictor of patient outcome than any of the individual modules (Table 1, HR 3.1; *P* = 0.0000021).

**Figure 3:**
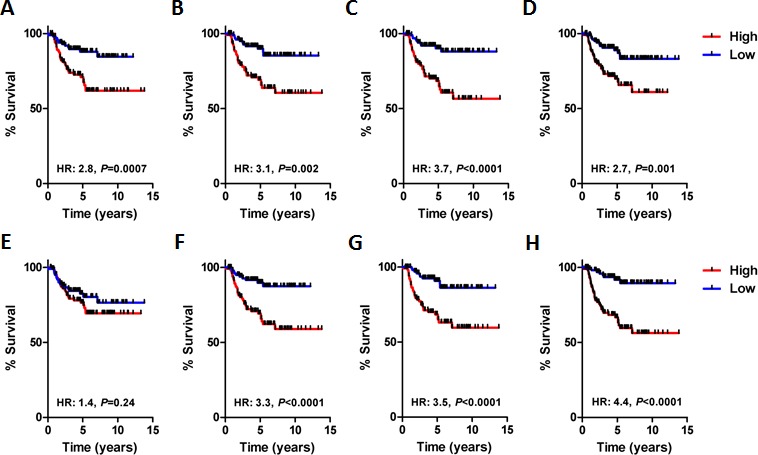
Survival analysis of each BLBC network module in the validation cohort (*n* = 211) **A.** Network module 0 (HR, 2.8; *P* = 0.0007, log-rank). **B.** Network module 1 (HR, 3.1; *P* = 0.002, log-rank). **C.** Network module 2 (HR, 3.7; *P* < 0.0001, log-rank). **D.** Network module 3 (HR, 2.7; *P* = 0.001). **E.** Network module 4 (HR, 1.4; *P* = 0.24, log-rank). **F.** Network module 5 (HR, 3.3; *P* < 0.0001, log-rank). **G.** Network module 6 (HR, 3.5; *P* < 0.0001, log-rank). **H.** Network module combination, average module index of all modules (HR, 4.4; *P* < 0.0001, log-rank).

We also stratified patients comprising the validation set into high- and low-risk groups based on the median module index value and completed survival analysis (Figure 3A-3G). In each case, with the exception of module 4, the individual module indices identified high- and low-risk patient populations with either poor or good survival characteristics respectively. The combination index robustly stratified the validation set patients into high- and low-risk group (Figure 3H, HR, 4.4; *P* < 0.0001). Indeed, the Kaplan-Meier estimate for 10-year survival in the low-risk groups was an excellent 90%, whereas in the high-risk group it was a dismal 56%. Hence, we concluded that the network modules were significantly associated with the outcome of patients with BLBC.

Importantly, the approach presented here varies substantially from other feature selection techniques in that protein interaction network data was used to identify the prognostic BLBC modules signature. To test whether identifying network modules from outcome-associated genes was a reasonable approach to select outcome-predictive genes, we calculated the difference in *P*-value scores (representing the negative logarithm of the *P*-value obtained from univariate Cox regression) of outcome-associated probe sets within the training and validation cohorts. On average, outcome-associated probe sets identified in the training cohort were less accurate in the validation cohort, resulting in a net decrease in average *P*-value score across all outcome-associated probe sets. However, we did not observe reduced *P*-value scores between the training and validation cohorts among outcome-associated probe sets that were included within the network modules (Supplementary Figure 1). Indeed, these data suggest that the identification of network modules from outcome-associated genes provides a reasonable step to reduce over-fit during training, thus providing a more robust means of predicting patient outcome.

### Individual BLBC modules are associated with specific biological pathways

We completed independent pathway analyses on each of the modules (Table 2). Modules 0, 4 and 5, which comprise a majority of poor outcome genes, were enriched in pathways related to cell stress, integrin signaling, and G-protein coupled receptor (GPCR) signaling, respectively. A dominant pathway predicted to be active in Module 0 was AP-1-meditated transcription, which included the module 0 genes *FOS*, *JUN*, *FOSB*, *ATF3*, *DUSP1*, and *EGR1*. Intriguingly, a role for the AP-1 transcription factor in BLBC has not previously been described, and might provide a therapeutic opportunity [27, 28]. Modules 1 and 2, which both comprise a majority of good outcome genes, were enriched in pathways relating to the function of T-cells and B-cells, respectively. It is likely that these modules measure the abundance and functionality of these various immune cell types within the tumor. Importantly, this finding is consistent with previous reports that T-cell infiltration in BLBCs is associated with good patient outcome [29]. These various modules might also be useful predictive markers to identify patients likely to respond to ‘immune-boosting’ therapies, such as CTLA4 and PD-1 blocking antibodies [30]. Module 6, which exclusively comprised genes related to good outcome, was enriched in apoptotic and NK cell pathways, once again highlighting the relationship between good outcome and immune infiltrate in BLBC. Notably, the presence of both NK cell and apoptotic pathway genes within module 6 might indicate that NK cells induce apoptosis within a subset of BLBCs.

**Table 2:** Pathway analysis of genes comprising each of the BLBC modules

**Module 0 – Stress pathways**	**FDR**
AP-1 transcription factor network(N)	<1.00e-03
HTLV-I infection(K)	<1.00E-03
ATF-2 transcription factor network(N)	<1.00e-03
ErbB1 downstream signaling(N)	<1.00e-03
Osteoclast differentiation(K)	<1.00e-03
**Module 1 - T cell**	
TCR signaling(R)	<1.00e-03
Primary immunodeficiency(K)	<1.00e-03
TCR signaling in naive CD4+ T cells(N)	<1.00e-03
T cell receptor signaling pathway(K)	1.50E-03
Cell adhesion molecules (CAMs)(K)	3.20E-03
Immunoregulatory interactions between a Lymphoid and a non-Lymphoid cell(R)	9.36E-03
**Module 2 - B cell**	
Fcgamma receptor (FCGR) dependent phagocytosis(R)	<1.00e-03
Complement cascade(R)	<1.00e-03
Signaling by the B Cell Receptor (BCR)(R)	<1.00e-03
Immunoregulatory interactions between a Lymphoid and a non-Lymphoid cell(R)	<1.00e-03
**Module 4 - Integrin signalling**	
Integrin signalling pathway(P)	<1.00e-03
**Module 5 - GPCR signaling**	
GPCR ligand binding(R)	3.00E-03
GPCR downstream signaling(R)	3.48E-02
**Module 6 – Apoptosis**	
Apoptosis(K)	<1.00e-03
Natural killer cell mediated cytotoxicity(K)	1.00E-03
Apoptosis(R)	5.71E-04

### Comparison of BLBC modules with other prognostic gene signatures

We compared the performance of the BLBC modules to multiple other prognostic gene signatures within the BLBC validation set. To this end we calculated and compared *P*-value scores for the Genomic Grade Index [5], NKI-70 signature [31], Oncotype DX^®^ score [3], CSR/Wound response signature [6], MS-14 signature [32], Glinsky stemness signature [33], Network module 2 [26] and the meta-PCNA signature described in Venet *et al.* [34] (Supplementary Figure 2A). Apart from the BLBC modules, only the Oncotype DX^®^ score was significantly associated with patient survival, albeit only marginally so. These findings corroborate the results of others, demonstrating that published prognostic molecular assays are not applicable to BLBC and highlight the challenges of this unique subtype [20]. We also compared *P*-value scores between the BLBC modules and additional signatures that were optimized to predict patient outcome specifically in ER-, TN, or BLBC [21, 22, 35, 36]. We observed that the BLBC modules yielded the highest *P*-value score, although the Hallett *et al.* and Teschendorff *et al.* signatures similarly displayed excellent performance (Supplementary Figure 2B). Overall, these data demonstrate that the BLBC modules offer an effective means to stratify BLBC patients into high- and low-risk groups. Notably, the BLBC modules were the most robust predictor of patient outcome among all surveyed predictors (*n* = 13).

### Network module TMA study

**Figure 4:**
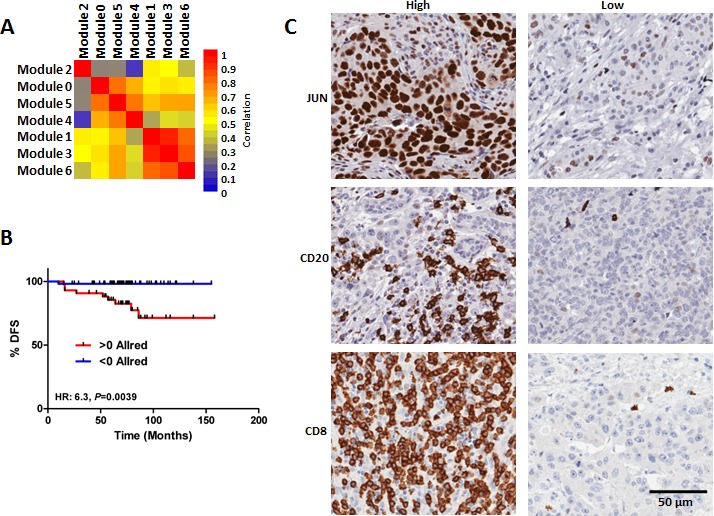
Validation of the BLBC network modules using and IHC TMA based approach **A.** Correlation of the module indices reveal relationship between modules 0, 4 & 5, modules 1, 3 & 6, respectively, as well as no relationship between module 2 and any other module. **B.** Survival analysis of the patients comprising the BLBC TMA stratified based on staining for JUN, CD8 and CD20. C) Representative photographs of high and low expression tumors for JUN, CD8 and CD20.

We next sought to capture the predictive information provided by the BLBC modules with a simple and clinically translatable IHC based assay. We first compiled a TMA from 102 FFPE BLBCs from Hamilton Health Sciences (HHS-cohort; characteristics summarized Supplementary Table 3). To reduce the number of features comprising the BLBC modules, we first measured the correlation between the indices obtained from each of the individual BLBC modules. The correlation between the module indices suggested that index scores calculated from modules 0, 4 and 5, and from 1, 3 and 6 were highly related, whereas the index scores from module 2 did not relate with index scores calculated from any other module (Figure 4A). Based on this data, we reasoned that each of the groups (modules 0, 4 and 5; modules 1, 3 and 6; and module 2) could each be represented with a single IHC measurement that could capture sufficient predictive information to be clinically useful. Taking into consideration the availability of high quality antibodies and our previous pathway analyses we selected JUN, CD8 and CD20 as representative markers of the 3 groups of modules. Each of the individual markers was evaluated using a modified Allred method; we subsequently calculated risk scores (BLBC modules IHC score) as the difference between JUN staining (high risk) and CD8 and CD20 (both low risk). We observed a robust relationship between the BLBC modules evaluated by IHC and patient survival (Figure 4B; HR 6.3; *P* = 0.0039, BLBC modules IHC score range [7 to −11]). The Kaplan-Meier estimate for 10 year survival in the low risk < 0 Allred score group was an excellent 98%, whereas in the > 0 Allred high risk group it was a much worse, 71% (Figure 4B). Representative staining for each antibody in high- and low-expressing tumor sections are shown (Figure 4C). In a univariate analysis that included grade, size, age, node and the BLBC modules IHC score, only node and BLBC modules IHC score were found to be statistically significantly related to patient outcome (Supplementary Table 4), whereas age, size and grade were not. In a multivariate model including node and BLBC module IHC score, both node (*P* = 0.004) and the BLBC modules IHC score (*P* = 0.0001) were significantly associated with patient outcome (Supplementary Table 5). Hence, we conclude that IHC staining for JUN, CD8 and CD20 captured similar outcome-associated information as to that measured by the BLBC modules, which is above and beyond that captured by standard clinical measurements.

## DISCUSSION

Few, if any, clinical variables can be used to successfully predict patient outcome in the context of BLBC. Therefore, we sought to identify a network based genomic predictor for patients with BLBC. We identified 7 BLBC network modules, which we tested and validated in an independent cohort of BLBC patients. Notably, the differences in outcome observed between patients predicted to have high- or low-risk BLBC was both large and statistically significant. In addition the network modules described were a more robust predictor of BLBC patient outcome than other published prognostic signatures evaluated which highlights the importance of incorporating molecular subtype into breast cancer biomarker development strategies [19].

Interestingly, pathway analysis of the individual BLBC modules suggested that each module was generally representative of a biological program that, depending on expression characteristics, was associated with either good or poor outcome. Modules 1, 2, 3, and 6, which together comprise the good outcome modules, were generally enriched in immune system processes. For example, module 1 genes were enriched in many T-cell pathways suggesting that the module 1 index identifies BLBCs rich in T-cell infiltrates. Indeed, the 3 biomarkers predictive of outcome in patients with BLBC included CD8, a marker of cytotoxic effector T-cells. Similarly, module 2 genes were enriched in many B-cell pathways, suggesting that this module serves as a biomarker of BLBCs with B-cell infiltrates. Interestingly, we did not observe significant correlation between Module 1 and Module 2 indices, suggesting that T-cell and B-cell infiltrates may be independently present within the stroma of individual BLBCs. Importantly, the presence of T-cell infiltrates and their relationship with outcome in BLBCs has been previously noted [22, 29, 37]. Similarly, observations linking a B-cell infiltrate with improved survival among TN breast cancer patients have also been reported [38-41]. Module 6 genes were also enriched in immune pathways related to NK mediated cytotoxicity and apoptosis, suggesting that module 6 may identify an NK cell infiltrate with tumoricidal activity. Taken together, these data suggest that immune infiltrates in BLBC are important determinants of patient outcome. Whereas CD8 T-cells and NK cells have previously been found to possess potent anti-tumor activity, other classes of lymphocytes or myeloid lineage cells including macrophages are thought to promote tumor progression and poor patient outcome (46). Indeed, Th1 and Th2 immune response pathways are reported to oppose and stimulate tumor development and progression in mouse models of breast cancer, respectively. For example, CD4 T cells can promote progression through interaction with tumor-specific macrophages, which subsequently stimulate the EGFR pathway leading to tumor metastasis [42]. Based on these reports, we suspect that our good outcome modules identify immune infiltrates and immune response pathways that function to produce an anti-tumorigenic microenvironment thereby leading to improved patient outcome.

Among the poor outcome modules (0, 4 and 5), we also observed enrichment for multiple signaling pathways and biological processes. Given that these modules are associated with poor patient outcome we think it is likely that these biological programs are associated with adverse tumor biology including progression and therapy resistance. Hence, their inhibition might provide therapeutic opportunities in BLBC. Targetable pathways enriched in Module 0 included the AP-1 and ATF-2 transcription factors, and ERBB1 (EGFR) downstream signaling. Whereas EGFR has been investigated as a possible therapeutic target in BLBCs [43], AP-1 and ATF-2 represent novel targets in BLBC. Intriguingly, the processes enriched in Module 5 genes were exclusively related to G-protein coupled receptor signaling (GPCR). GPCRs are widely regarded as highly drugable targets, although GPCRs have not been traditional targets for anti-cancer drug development [44]. Traditional GPCR targeted drugs include those that interfere with neurotransmitter signaling, such as dopamine and serotonin receptor antagonists and are widely prescribed to treat mood disorders [45]. Recent reports suggest that many of these drugs display anti-cancer activity in pre-clinical models, including breast cancer models [46, 47]. Our work supports these findings and provides additional evidence that GPCR signaling drives aggressive tumor behaviour and represents a therapeutic target in BLBC.

There are several limitations of the work described herein. All of our conclusions are based on the analysis of retrospective data, which limits its clinical value. We demonstrated the predictive capacity of the BLBC modules in an independent gene expression cohort, as well as with an exploratory 3-biomarker IHC test in a local archival FFPE cohort. However, a true estimate of the clinical usefulness of the BLBC modules will require additional validation in clinical trial samples, or completion of a prospective clinical trial examining the capacity of the BLBC modules to accurately identify low- and high-risk BLBC patients [48]. In addition, it is not clear whether the predictive capacity of the BLBC modules is a consequence of measuring the natural progression of BLBCs or predicting BLBC response to anti-cancer therapy. Whereas the majority of the training patients were chemotherapy naive, suggesting that the BLBC modules are prognostic, the validation cohort comprised a majority of chemotherapy treated patients (Supplemental Table 6). Hence, we cannot make precise conclusions about the prognostic and predictive capacities of the BLBC modules and acknowledge that their association with patient outcome may contain both prognostic and predictive elements.

As mentioned above, no robustly validated biomarker test exists to predict BLBC patient outcome. Here we present a network based and genomics driven approach to identify outcome-associated BLBC network modules. Moreover, we also validated the BLBC network modules using an IHC based surrogate assay in an additional series of BLBCs. Given the strong relationship observed between the BLBC modules and patient outcome as well as the widespread availability of IHC, our findings if validated, could be rapidly implemented into the clinic as a means to spare low risk BLBCs patients from aggressive therapy as well as target aggressive therapies to those patients with high risk tumors in a timely fashion.

## MATERIALS AND METHODS

### Assembly of datasets

A diagram summarizing the analytical strategy and the identity of the training and validation cohorts is included (Supplementary Figure 3). For the training set, we analyzed the gene expression profiles *in silico* of 5 independent external datasets, obtained using Affymetrix HG-U133A GeneChip arrays, which have been deposited in the Gene Expression Omnibus (GEO); accession numbers GSE1456, GSE2034, GSE3494, GSE6532, and GSE7390 and comprise a total of 1077 samples (summarized Supplementary Table 1).

For the validation set, we analyzed the gene expression profiles *in silico* of 5 publically available datasets obtained using Affymetrix HG-U133plus2.0 GeneChip arrays. These profiles were deposited in the Gene Expression Omnibus (GEO) (accession numbers GSE20685, GSE21653, GSE16446, GSE19615 and GSE9195) and comprise a total of 905 samples with accompanying clinical follow-up data (summarized in Supplementary Table 2).

All samples used for our study were normalized with frozen Robust Multi-array Analysis (fRMA), a procedure that allows one to pre-process microarrays individually or in small batches and to then combine the data into a single dataset for further analysis as previously described [49]. Thereafter we used the DWD (Distance-Weighted Discrimination) [50] method to remove technical variation from the datasets that were to be combined for future analysis. After combining all datasets, Spearman correlation coefficients for pair-wise comparisons of samples using 62 house-keeping probe sets were computed, and only samples exhibiting a correlation higher than 0.95 with at least half of the dataset were selected for further classification. The latter filtering method yielded datasets comprising 995 and 894 human breast tumor sample transcript profiles for training and validation respectively.

### Tumor molecular subtype assignment

All tumors from the independent datasets were classified as basal-like, HER2+, luminal A, luminal B, claudin-low, normal-like or apocrine by assigning them to the standardized centroid of the subtype to which they had the highest Spearman rank correlation [11, 12, 51-53]. The correlation was computed using 710 intrinsic genes as previously described [51]. Reference samples used to calculate standardized centroids for the apocrine subtype were taken from Farmer et al [53] and for the basal-like, HER2+, luminal A, luminal B, claudin-low and normal-like subtypes from Prat et al [54]. Gene symbols were used to match the probes and genes with Gene Symbol names. These data were averaged and samples were median-centered for all datasets prior to subtype assignment. Detailed information, including clinico-pathological features of the tumors are included in Supplementary Table 6.

### Network analysis

BLBC modules identification was implemented using the Cytoscape Reactome FI plug-in [26]. Briefly, outcome associated probe sets were mapped to unigene ID and subsequently mapped to nodes in Reactome [25]. Weights were assigned to edges connecting interacting nodes based on the absolute value of the Pearson correlation co-efficient of expression. Markov Clustering (MCL) was implemented to identify network modules, and we selected modules comprising at least 8 nodes with average Pearson correlation of at least 0.25.

### Network index and signature score calculation

We calculated either module indices or signature scores as the difference between the geometric means (of Log_2_ expression) of the poor and good outcome associated genes, respectively, similar to previous reports [35, 55]. This occurred as follows:

$$\frac{\sqrt[{nP}]{x_{1}•x_{2}•...x_{n}}}{n_{P}}-\frac{\sqrt[{nN}]{x_{1}•x_{2}•...x_{n}}}{n_{N}}$$

Where *x* is the log_2_ expression, *n* is the number of probe sets, *P* is the set of probes reported to be associated with poor outcome, and *N* is the set of probes reportedly associated with good outcome.

### Assembly and analysis of tissue microarray (TMA)

Formalin fixed paraffin embedded (FFPE) blocks of triple negative (TN) invasive breast cancer from the pathology archive of Hamilton Health Sciences (HHS) for the years 2005 to 2009 were collected with institutional review board approval. Each tumor was evaluated for the expression of CK5/6 and EGFR by standard immunohistochemical (IHC) techniques. Those tumors identified as TN and positive for either CK5/6 or EGFR were deemed BLBCs as previously described [14]. The pathological staging, treatment and clinical outcome data for each patient was abstracted from the patient clinical files by an experienced clinical research associate. Patient tumors were excluded if they were locally advanced at presentation, had high nodal status (≥N2) or if the patient did not receive standard of care management. A total of 102 such tumors were identified. Three 0.6mm cores were taken from the FFPE blocks and used for TMA construction. Each slide was stained with antibodies against CD8, CD20, and JUN, and quantified using the Allred method by an individual blinded to clinical outcome [56]. The highest Allred score for each of the three cores was used to calculate the Allred score for each sample. To calculate an Allred score based on these 3 markers we used the formula Allred^JUN^-Allred^CD8^-Allred^CD20^. We confirmed the reproducibility of this approach by having a second individual blinded to outcome re-evaluate the scoring approach (Pearson correlation: 0.84, *P* = 0. 6.8E-29).

### Statistical analysis

Cox-regression analysis was completed in R using the *CoxPH* package. Kaplan-Meier analysis was completed using GraphPad Prism^TM^ 5; *P*-values less than 0.05 were taken to indicate statistical significance.

## SUPPLEMENTARY MATERIALS FIGURES AND TABLES




