# Supplementary material for: Identification and evaluation of network modules for the prognosis of basal-like breast cancer
Source: Oncotarget. 2015 May 8;6(19):17713–24. doi: 10.18632/oncotarget.4034 (PMC4627340; doi:10.18632/oncotarget.4034)
Supplement: Supplementary file 1 [file oncotarget-06-17713-s001.pdf]

Identification and evaluation of network modules for the prognosis of basal-like breast cancer

Supplementary Material

A

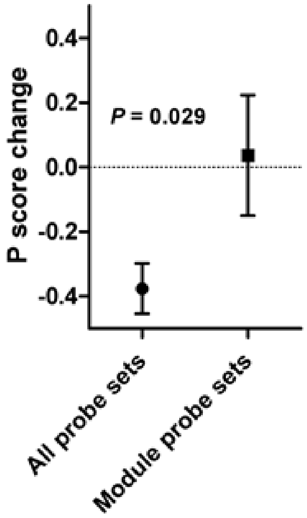

Supplementary Figure 1: A) Comparison of *P*-value scores between unselected outcome associated probe sets and network module selected outcome associated probe sets in the training and validation cohorts

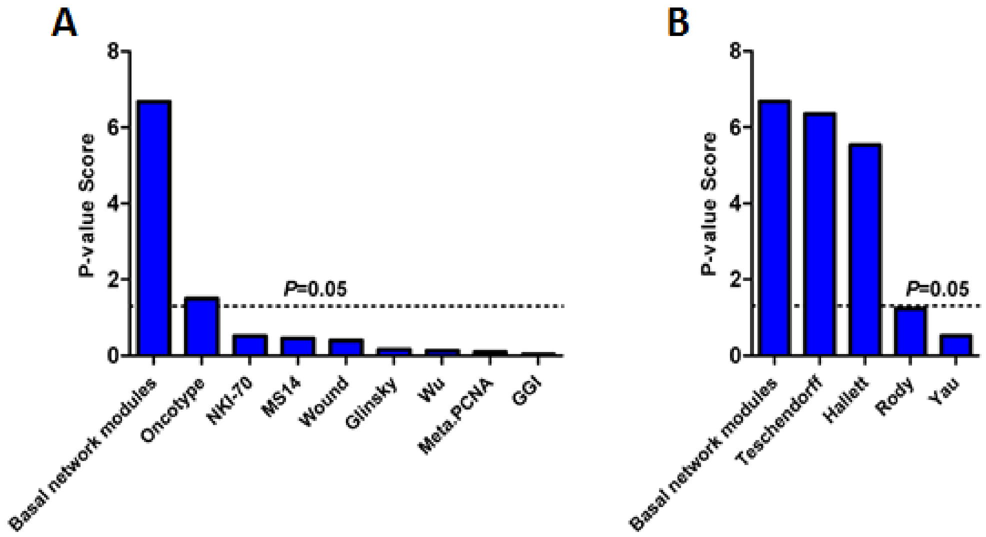

Supplementary Figure 2: Comparison of multiple outcome-associated gene signatures in BLBC. A) *P*-value scores (-log[p-value cox-regression]) for several established and well validated published gene signatures. B) *P*-value scores for outcome associated gene signatures developed specifically for ER-, TN, and BLBC tumors.

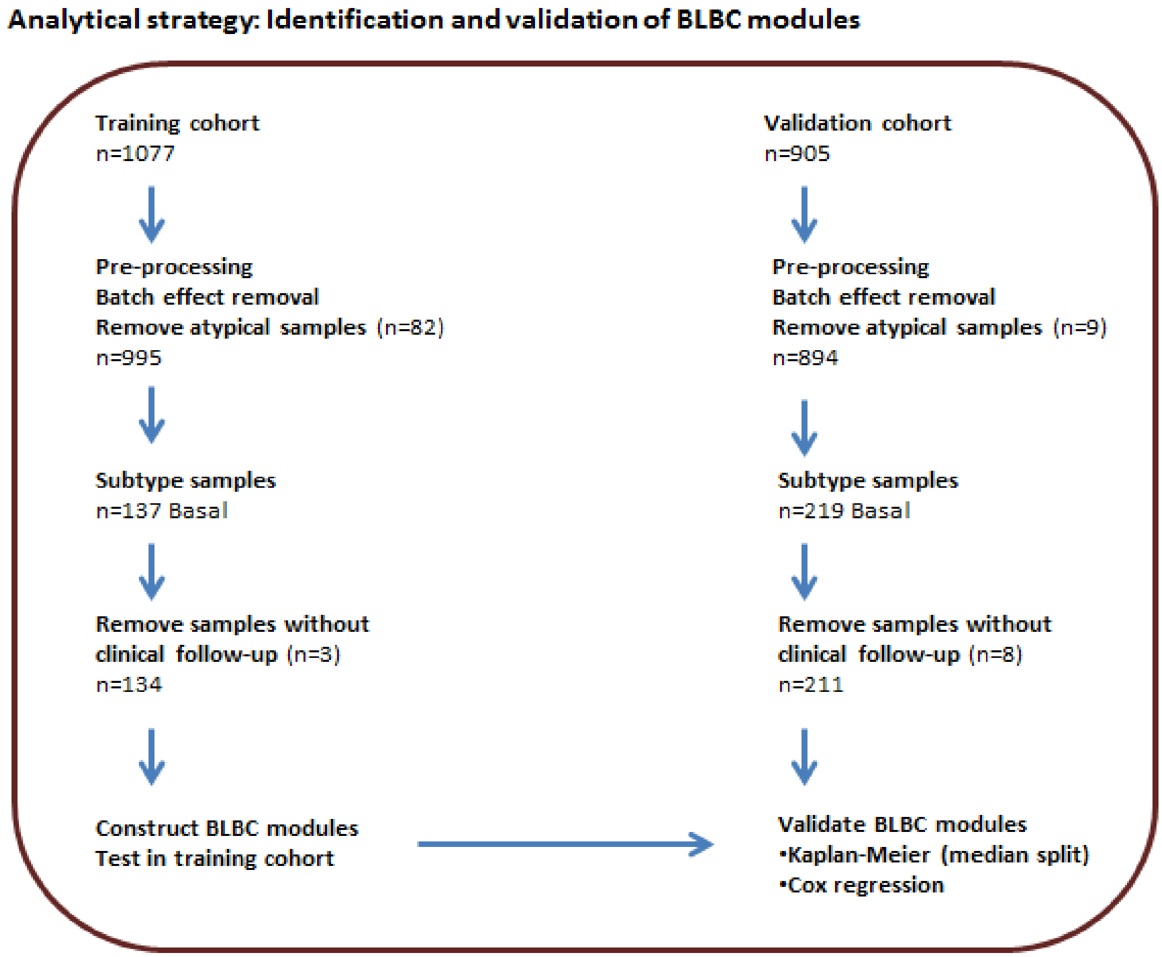

Supplementary Figure 3: Analytical strategy describing training and validation cohorts, as well as BLBC module identification and validation
